# A Stable Biotin-Streptavidin Surface Enables Multiplex, Label-Free Protein Detection by Aptamer and Aptamer-Protein Arrays Using Arrayed Imaging Reflectometry

**DOI:** 10.3390/s20205745

**Published:** 2020-10-10

**Authors:** Alanna M. Klose, Benjamin L. Miller

**Affiliations:** Department of Dermatology, University of Rochester Medical Center, Rochester, NY 14642, USA; alanna_klose@urmc.rochester.edu

**Keywords:** biotin, streptavidin, aptamer, protein, microarray, reflectometry

## Abstract

While label-free multiplex sensor technology enables “mixing and matching” of different capture molecules in principle, in practice this has been rarely (if ever) demonstrated. To fill this gap, we developed protocols for the preparation of mixed aptamer-protein arrays on the arrayed imaging reflectometry (AIR) sensing platform using streptavidin as a common attachment point for both biotinylated proteins and aptamers. Doing so required overcoming the noted instability of dried streptavidin monolayers on surfaces. After characterizing this degradation, stable surfaces were obtained using a commercial microarray product. Microarraying through the layer of stabilizer then provided mixed aptamer-antibody arrays. We demonstrate that sensor arrays prepared in this manner are suitable for several probes (thrombin and TGF-β1 aptamers; avi-tagged protein) and targets.

## 1. Introduction

Array-based detection strategies typically rely on a single type of capture probe for all analytes [1]. For example, arrays designed to detect and quantify proteins in a sample may consist entirely of antibodies specific for each target, while those intended to assay nucleic acids may consist entirely of target-specific DNA probes. However, since each spot (or detection area) on a sensor array operates independently, there is no fundamental barrier against mixing and matching different types of capture molecules on the array. Doing so would allow the sensor array to employ the best available probe for each target, rather than limiting choices to a single probe class. In practice, however, integrating aptamer and antibody (or other protein) probes on an array is challenging due to differences in immobilization chemistry. In the interest of expanding the versatility of optical sensor arrays, we initiated a study to determine conditions suitable for mixing two of the most common probe types: aptamers and antibodies. We have done this using arrayed imaging reflectometry (AIR), a label-free optical sensor well established for the detection of biomolecules [2]. This technology uses s-polarized HeNe laser light at a 70.6° incident angle to the chip surface. The surface film thickness (SiO_2_, adhesion chemistry, and deposited capture molecules) is tuned based on the incident wavelength, angle, and polarization to create near-perfect destructive interference of light incident with the surface. This antireflective condition is disrupted by changes in optical path length caused by target molecules binding to any probe spot. The reflected light is imaged by a CCD camera, quantified, and converted to thickness by an experimentally derived reference curve defining the relationship between light intensity and thickness [3]. Experimentally, the deposition of probe molecules onto the chip surface does not always result in a perfectly antireflective condition for each probe type; this leads to slight differences in thickness between probe types, which is accounted for by using a control chip to identify base probe thickness. 

The first critical question when preparing a mixed-probe array is that of surface attachment chemistry. In past work, we have consistently used an amine-reactive surface for covalent immobilization of proteins and peptides [3]. Preliminary attempts to employ this chemistry with amine-terminated nucleic acids were unsatisfactory, and so we turned to deposition of pre-formed complexes of streptavidin with biotin-tagged oligonucleotides [4]. This method inherently results in wastage of probe oligonucleotide and is also operationally undesirable given the requirement for separate preparation of each streptavidin-probe complex prior to microarray fabrication. Therefore, we examined the preparation of uniform streptavidin films. 

Many sensor platforms utilize the specific, “near-covalent” affinity of biotin and streptavidin to create attachment points for capture probes or detection labels [5]. The strong affinity between biotin and streptavidin, avidin, or neutravidin is well characterized in aqueous conditions [6] but is less understood at air-liquid interfaces. Likewise, few studies have explicitly tested the durability of streptavidin (and related protein) films through cycles of drying and rewetting. Many sensor platforms using dry streptavidin films require protective coatings to prevent degradation of the film [7,8]. For example, the drying-induced degradation of streptavidin from a biotin-containing alkylthiolate monolayer on gold has been characterized by atomic force microscopy (AFM), and suggests loss of streptavidin from the biotin surface after drying with nitrogen gas. Degradation of the film was mitigated by treatment with a trehalose solution prior to careful drying with humidified air [7]. 

The biotin-streptavidin surface has the potential to be a powerful tool for other planar, microspotted sensing platforms because it enables the attachment of any detection probe conjugated with a biotin tag. However, prior to the work we describe herein, it was unknown whether the degradation of dried streptavidin films would occur on other sensing platforms with different linker chemistry, and, if so, what the physical nature of the degradation might be. Here we describe the growth, degradation, and preservation of a biotin-streptavidin (B-SA) surface on an amine-reactive silicon chip for protein detection by AIR. Preservation of the B-SA surface was accomplished using a commercial microarray stabilization product (StabilCoat Plus^®^). Remarkably, microarray printing of biotinylated probes through the stabilization layer was efficient, yielding viable AIR microarrays consisting of biotinylated protein probes, aptamer probes, or both. This work broadens the utility of streptavidin films in sensing and provides the first example, of which we are aware, of a mixed aptamer/protein label-free microarray.

## 2. Materials and Methods

Materials: Silicon AIR substrates with amine-reactive surfaces (chips) were purchased from Adarza BioSystems, Inc. Amine-PEG_11_-biotin and amine-PEG_4_-methyl were purchased from Thermo Fisher. Streptavidin, avidin, anti-fluorescein (FITC) (goat polyclonal), bovine serum albumin (BSA), and human IgG were purchased from Rockland Immunochemicals. Sulfo-NHS-LC-LC-biotin was purchased from Thermo Fisher. StabilCoat Plus^®^ was purchased from Surmodics. All aptamers were synthesized and underwent initial purification (discussed below) by Integrated DNA Technologies. Thrombin from human plasma and biotin-conjugated BSA were purchased from Sigma-Aldrich. Recombinant human TGF-β1 produced in CHO cells was purchased from PeproTech. Biotinylated Recombinant Human Fcγ RIIIA/CD16a (F176) His-tag Avi-tag and anti-Interferon Gamma (IFNγ) were purchased from R&D Systems. Poly(N-isopropylacrylamide)-co-allylamine (PNIPAM-coAA) particles were synthesized and biotinylated in our laboratory following previously published protocols [9].

Optimized Growth and Protection of Biotin-Streptavidin Surface: The amine-reactive chips were first mounted onto adhesive strips (combs) at appropriate spacing for a 96-well plate. They were then incubated in a 30% amine-PEG_11_-biotin and 70% amine-PEG_4_-methyl (total of 1.3 mM linkers) in 1x PBS solution (PBS:137 mM NaCl, 2.7 mM KCl, 10 mM Na_2_HPO_4_, and 1.76 mM KH_2_PO_4_-H_2_O, pH 7.4) overnight, with agitation on a rotating platform shaker, at room temperature (Figure S1). Next, chips were washed in an assay wash buffer (AWB: mPBS (150 mM NaCl, 10 mM Na_2_HPO_4_, 10 mM NaH_2_PO_4_) with 0.005% tween-20, pH 7.2) for 5 min before incubation in 40 µg/mL streptavidin in PBS for 1 h, shaking, at room temperature. After batch-rinsing the chips for 5 min in AWB, they were incubated in a 1% solution of StabilCoat Plus^®^ in 18 MΩ-cm water produced by a Barnstead Nanopure II purification system (Nanopure^TM^) for 20 min before being spun dry for 1 min at 500 RPM after being attached to the rotating platform of a wafer polisher (Ecomet 4, Buehler, IL, USA) (Supplementary Video S1).

Other Protective Layers on the Biotin-Streptavidin Surface: The growth of the B-SA surface proceeded as described above, but instead of incubating the chips in a 1% solution of StabilCoat Plus^®^, the chips were incubated in either 1% or 3% solutions of trehalose in Nanopure^TM^ water, or a 5%, 10%, or 50% solution of StabilCoat Plus^®^ in Nanopure^TM^ water. Each of these protective coatings was applied in a 20-min incubation, then removed either by spin-drying as described above, or by briefly drying under a stream of dry N_2_ gas. In the instances where chips were left to dry on the laboratory benchtop, they were briefly shaken to remove excess liquid, then placed face up on the counter. Any remaining liquid pooled into the center of each chip and slowly evaporated. The laboratory air was measured to be around 16% relative humidity (RH) and 23 °C during the time that these experiments were conducted.

Aptamer Sequences: Aptamer sequences were selected from the Aptagen “Apta-index” database based on literature reports [10], and synthesized with 5’ biotin and polyT(20) spacer modifications. Thrombin aptamers were sufficiently pure as obtained from the supplier to be used following desalting, while the TGF-β1 aptamer was desalted and further purified by HPLC. Note that the TGF-β1 aptamer incorporated phosphorothioate bases at positions indicated by asterisks.

Thrombin 15-mer: 5′-biotin-polyT(20)-GGTTGGTGTGGTTGG-3′. 

Thrombin 29-mer: 5′- biotin- polyT(20)-AGTCCGTGGTAGGGCAGGTTGGGGTGACT-3′. 

TGF-β1: 5′-biotin-polyT(20)-CGCTCGGCTTCACGAGATTCGTGTC*GTTGTGTC*C*TGTA*C* C*C*GC*C*TTGA*C*C*A*GTC*A*C*TC*TA*GA*GC*A*TC*C*GGA*C*TG3′.

Microarray Spot Deposition: 10 µL volumes of all solutions to be printed were pipetted into individual wells of a 384-well source plate to be used for printing. Biotinylated proteins were diluted in PBS. Aptamers were diluted in aptamer immobilization buffer (5 mM Tris-HCl, 1 M NaCl, 0.5 mM EDTA, pH 7.4), and refolded using a thermocycler (5 min at 90 °C, 15 min linear decrease in temperature to 25 °C) immediately before dilution in the 384-well plate. Either an S3 or SX sciFLEXARRAYER (Scienion A.G.; Berlin, Germany) with a PDC60 or PDC70 capillary nozzle was used to print probes onto chips mounted onto adhesive strips inside a controlled humidity chamber at 73% ± 4% RH. Droplets were between 250 and 350 pL in volume (depending on the nozzle used), as measured by the instrument. After deposition, spots were analyzed with an overhead camera to ensure their uniformity and to observe their interaction with the surface. The chips remained in the humidified chamber for 30 min to enable full immobilization of biotinylated materials before being removed.

Target Detection: The microarrayed chips were removed from the humidified microspotting chamber and immediately placed into wells of a 96-well plate containing a solution of 0.1 mg/mL D-biotin in AWB for 5 min to prevent smearing of any unadsorbed probes onto nearby streptavidin surface. The chips were then incubated in 1% BSA in AWB for 30 min to block the background from nonspecific adsorption. The chips were washed with AWB for 5 min before being transferred to incubation wells that had been pre-blocked with 1% BSA in AWB before being refilled with a dilution series of thrombin from 1 µg/mL to 1 ng/mL in 10-fold decrements diluted in aptamer binding buffer (ABB; 50 mM Tris-HCl, 140 mM NaCl, 1 mM MgCl_2_, 1% BSA, and 0.05% Triton X-100, pH 7.4), or a dilution series of TGFβ1 (1 µg/mL, 500 ng/mL, 100 ng/mL, 50 ng/mL, 10 ng/mL, and 1 ng/mL in ABB). The chips were incubated in target overnight at 4 °C on a shaker. Chips were then rinsed twice (10 min each rinse) with AWB. After a final rinse in Nanopure^TM^ water, chips were dried under a stream of nitrogen gas. Control chips were exposed to ABB during the overnight incubation.

Imaging with Arrayed Imaging Reflectometry and Data Analysis: All microarrays were imaged on a prototype AIR imaging system (Adarza BioSystems, St. Louis, MO, USA). Images were acquired for each substrate using custom in-house instrument control software at 500 or 1000 ms exposure times. Spot intensities were measured using ImageJ (v. 1.51j8) [11], and converted to thicknesses using a best-fit line to an experimentally derived reflectance model [3]. Average measurements were composed of at least 4 replicate spots on each chip, across three chips, with errors represented by the standard deviation of all replicate probe spots. On-chip normalization probes, consisting of biotin-conjugated BSA, biotin-PNIPAM-co-AA, or biotin-conjugated anti-INFγ, depending on the array (Figure S6), resist binding by target protein, and are used to account for any variation in initial surface film thickness or reactivity between chips. The intensity-converted thickness values of the normalizing spots are subtracted from the probe values which they flank to produce the reported “normalized thickness” (Å) values (Figure S2). Control chips, which undergo all processing steps in parallel with sample chips, are not exposed to target molecules. These are therefore used to determine baseline probe thicknesses at 0 ng/mL concentration and are used as blank values for curve fitting and limit of the blank (LOB) calculations. A 4-parameter logistic fit was applied to the TGFβ1 and thrombin dilution curves using a Matlab (Mathworks R2020a) script, which was also used to calculate the limit of the blank (LOB), limit of detection (LOD), and 95% confidence intervals [12]. LODs were compared between assays using a Student’s t-test of unequal variances. The degrees of freedom (df) were calculated by taking each assay as a whole, including every replicate at each sample dilution (n = 119).

Atomic Force Microscopy: The surface topography of dried microarrays was analyzed with a Solver Next atomic force microscope (NT-MDT, Moscow, Russia). Both 80 and 30 μm scans were conducted in tapping mode, with a pixel density of 256 in both x and y dimensions.

Spectroscopic Ellipsometry: The thickness of dried substrates was measured with an alpha-SE ellipsometer (J.A. Woollam, Lincoln, NE, USA.) and analyzed with CompleteEASE^TM^ software (v. 3.60) using a model for a layer of silicon dioxide on a silicon substrate with a maximum mean squared error (MSE) of 20. MSE is defined as in Equation (1) [13], where N = Cos(2Ψ), C = Sin(2Ψ), S = Sin(2Ψ)Sin(Δ), n corresponds to the number of wavelengths analyzed, m is the number of parameters in the fit, and Ψ and Δ are standard spectroscopic ellipsometry parameters defining the reflectivity and phase of the measurement.

(1) $$MSE=\;\sqrt{\frac{1}{3n-m}\overset{n}{\underset{1}{\sum }}[{\left(N_{E_{i}}-N_{G_{i}}\right)}^{2}+{\left(C_{E_{i}}-C_{G_{i}}\right)}^{2}+{\left(S_{E_{i}}-S_{G_{i}}\right)}^{2}]}\;\times \;1000$$

## 3. Results and Discussion

### 3.1. Generation of the B-SA Surface

**Figure 1:**
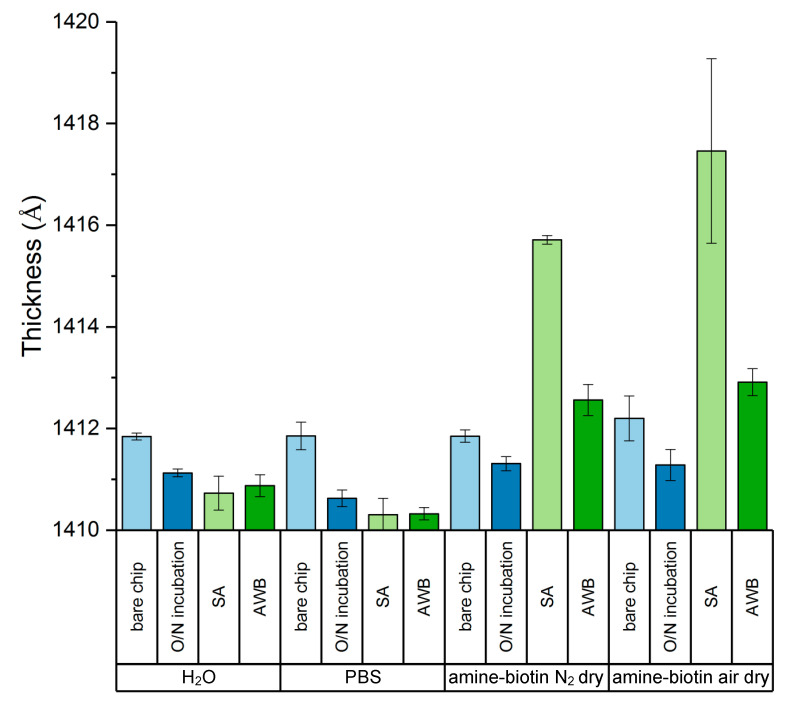
Growth of the biotin-streptavidin lawn. The overnight (O/N) incubation conditions were H_2_O, PBS, or a mixture of amine-PEG_11_-biotin and amine-PEG_4_-methyl. Thicknesses are the averages of three chips measured via ellipsometry and fit to a model of silicon dioxide layer on silicon substrate after each step of the growth process. Error bars represent the standard deviation of thickness measurements between three replicate chips. All chips were washed with Nanopure^TM^ water and dried with a nitrogen gas stream after each incubation and prior to ellipsometry except after streptavidin where one set of chips (indicated above) was allowed to dry in ambient air on the laboratory benchtop. Streptavidin only builds onto the chips exposed to amine-biotin, and a significant loss of surface thickness is observed after drying and rewetting with assay wash buffer (AWB) after either drying condition.

The B-SA surface was generated by an overnight incubation of chips in a mixture of amine-PEG_11_-biotin and amine-PEG_4_-methyl, followed by a one-hour incubation in streptavidin (Figure S1). Changes in thickness of the chip film at each stage of the B-SA surface preparation were characterized by spectroscopic ellipsometry and fit using a model of a silicon dioxide layer over a silicon substrate (Figure 1). As we have previously reported, exposure of the silanized (amine-reactive) surface to aqueous conditions causes desorption of silane and concomitant loss of thickness [14] (“H_2_O” and “PBS” sections of Figure 1). Further treatment of the surface with a molecule such as a protein stabilizes the silane. While no measurable increase in film thickness was observed after treatment with the amine-PEG_11_-biotin/amine-PEG_4_-methyl mixture (potentially due to the PEG chains from a sparse monolayer lying flat on the surface after drying), only chips treated in this way bound streptavidin. Chips that had been incubated in water or PBS overnight were completely deactivated and unable to adsorb or chemisorb streptavidin. However, there was a four to six Ångstrom build of streptavidin onto the surfaces exposed to the amine-biotin mixture. This indicates that the B-SA surface grew as expected. Since others have found that similar B-SA surfaces have degraded upon drying with a stream of nitrogen gas [7], a set of chips were instead allowed to gently dry on the laboratory benchtop. The chips dried in this manner had a greater variability in streptavidin thickness than the chips dried under a stream of dry nitrogen gas, but the average build of streptavidin was similar. Incubation of the dried chips in AWB, followed by a rinse in Nanopure^TM^ water and drying by a stream of nitrogen gas, caused a substantial decrease in thickness. This thickness loss was the first indication that the B-SA surface was not stable after drying either by nitrogen, or with the gentler benchtop dry. 

### 3.2. Characterization of the Dried and Rewetted Biotin-Sa Surface via AIR and AFM

**Figure 2:**
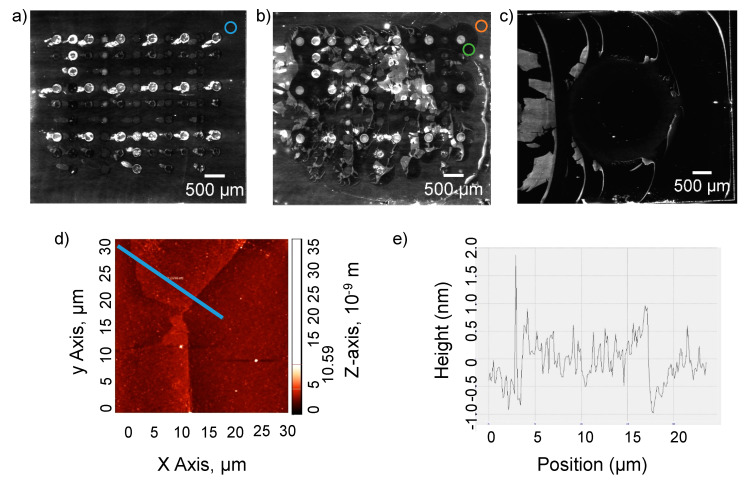
Characterization of the biotin-SA surface degradation after drying, probe deposition, and rewetting. Chips were either dried by a stream of nitrogen gas (**a**) or dried on the benchtop (**b–d**) after growth of the biotin-SA surface and prior to probe deposition in a humidified chamber. Chips were incubated in the chamber for 30 min after deposition, followed by incubation in target detection solutions (**a**,**b**,**d**) or 5 min in AWB (**c**), rinsed with Nanopure^TM^ water, and dried via a stream of N_2_ prior to imaging. Panels (**a–c**) are arrayed imaging reflectometry (AIR) images, while panels (**d**) and (**e**) are the result of analysis by atomic force microscopy (AFM). The array probe morphology was undesirable on chips where the B-SA surface had been dried under a stream of N_2_ (**a**). The surface appears to peel away around each probe in the array (250–350 pL droplets) (**b**). The median intensity of the chip surface within the colored circles was measured with Image J; Blue: 8314, Orange: 16942, Green: 6837 (**a**,**b**). The peeling phenomenon scales with the size of the deposited probe (1 µL droplet, pipetted by hand) (**c**). Morphological analysis by AFM suggests a peeled layer folded over on itself (**d**,**e**).

To gain a better understanding of what might be happening with the unprotected surface upon drying and rewetting, chips were imaged by AIR after probe deposition and incubation in target detection solutions. The B-SA chips that had been dried with nitrogen gas prior to probe deposition have undesirable probe morphology that looks as if excess probe streaked off onto the surrounding chip surface upon rewetting (Figure 2a). However, we were struck by the observation that chips allowed to dry in ambient laboratory air on the benchtop prior to probe deposition have a morphology resembling peeling (Figure 2b). Perhaps both surfaces underwent peeling upon rewetting, but the nitrogen dry was so harsh that all of the surface completely peeled and detached while the benchtop-dried surface partially peeled and remained attached. This idea is supported by measuring the median intensity of selected regions of each chip image. The blue circle measuring the background of the nitrogen dried chip has a median intensity of 8314 (Figure 2a). The undisturbed background of the benchtop dried chip (orange circle) has an intensity of 16,942, but the background that seems to have peeled away on the benchtop dried chip (green circle) has an intensity of 6837 (Figure 2b), which is similar to the background of the nitrogen dried chip. This behavior was observed at a larger scale via AIR after manually pipetting 1 µL of PBS onto the benchtop-dried B-SA surface (Figure 2c). This decrease, and a partial “peeling” effect of the SA layer upon rewetting, was confirmed by AFM analysis (Figure 2d). The bright areas of the degraded surface are approximately twice as thick as the neighboring dark areas, which suggests a peeled surface folded over on itself (Figure 2e).

Other studies have reported evidence that proteins become denatured or partially unfolded when they contact an air-water interface (AWI) [15,16]. Streptavidin denaturation was observed at the AWI during single particle electron cryo-microscopy (cryo-EM) reconstruction, and protein molecules at the interface were damaged compared to the ones that adsorbed to a graphene grid and were not exposed to the AWI [16]. In our work, streptavidin tetramers are constrained by one or more of their subunits via the biotin linkers covalently attached to the chip. Immobilization demands that the streptavidin proteins experience hostile AWI conditions, as water is rapidly removed from the chip surface. As the surface dries, the streptavidin may denature or change conformation, thus losing affinity for biotin. Forces in the range of 100–400 pN on a single subunit of streptavidin have been shown to disrupt the protein’s affinity for biotin [17]. In our work, even if the dried streptavidin proteins changed conformation, or denatured upon drying and lost affinity for biotin enough to desorb from the biotin surface, it is not clear how individual SA tetramers would remain associated with each other in such a way as to produce the observed peeling phenomenon. Whey protein films are produced by thermal denaturation of proteins to produce irreversible aggregates which crosslink [18]. However, such an extreme case of irreversible denaturation occurs at high temperature and is not likely to be occurring here. Inspection of the array image in Figure 2b suggests the peeling effect is less pronounced within the arrayed probe spots, potentially due to stabilization by the deposited biotin-conjugated proteins.

Further supporting the hypothesis that the degradation is caused by exposure to the AWI is the knowledge that other groups have reported using protective coatings to preserve the stability of their streptavidin surfaces. These streptavidin surfaces were all produced on different material substrates with different linking chemistry, and each one required a protective coating, typically a dilute trehalose mixture [7,8], or other proprietary coating [19] to prevent degradation of the surface. This supports the idea that the point of weakness is between the biotin and the streptavidin rather than between the biotin linker and the substrate surface. Presumably, the trehalose helps to hold water in the surface and reduces the effects of the AWI on protein denaturation. Surfactants are used in cryo-EM to achieve a similar “cover slip” effect [15].

### 3.3. Optimization of Protective Coating and Preparation of Microarrays

Although we were not aware of any prior reports of microarray spotting through a protective coating, this nevertheless seemed an appropriate approach to take. To prevent the degradation of the B-SA surface during drying and rewetting, we incubated the B-SA chips in solutions of trehalose or StabilCoat Plus^®^ (a commercial microarray stabilizing product) in Nanopure^TM^ water prior to drying. The 1% and 3% trehalose solutions successfully mitigated surface degradation, but also produced unacceptable drying inconsistencies and poor probe morphology (Figure S3). This was unexpected, given the successful use of trehalose solutions with other sensor systems [7,8]. The observed spot artifacts likely arise during the dissolution of the protective coating when the protein solutions are microarrayed onto the surface. In contrast, the initially tested concentration of 50% StabilCoat Plus^®^ prevented degradation of the B-SA surface, but resulted in diffuse probe morphology and smearing (Figure S3). We concluded that the high concentration of StabilCoat Plus^®^ prevented the probes from diffusing through the protective coating and adhering to the streptavidin surface below. 

**Figure 3:**
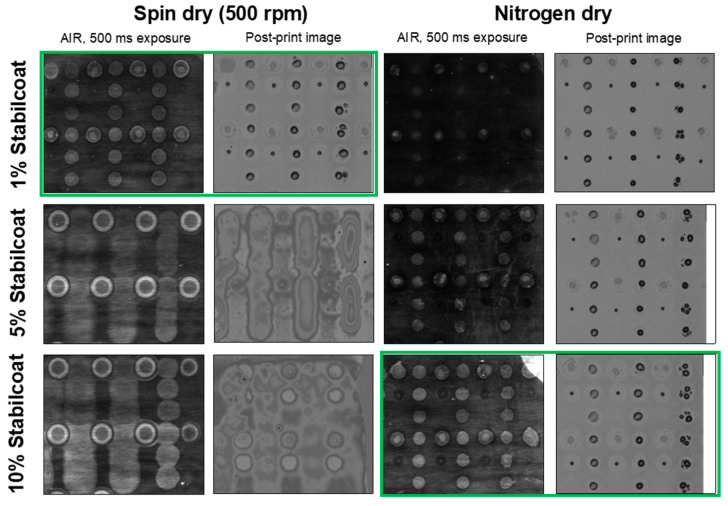
Optimization of protective coating conditions. The protective layer must be thin enough for probes to dissolve through cleanly during the printing process without substantial radial diffusion, but thick enough to prevent degradation of the biotin-SA surface. Printer head-cam and AIR images are shown for comparison. The spin-dry procedure requires a lower percentage of StabilCoat Plus^®^ in Nanopure^TM^ (1% is optimal) than drying under a nitrogen stream (10% is optimal). Optimal conditions in each case are highlighted in green. Arrays consist of biotin anti-FITC flanking thrombin 29-mer (top 3 rows) and 15-mer (bottom 3 rows).

While trying lower concentrations of StabilCoat Plus^®^ (1%, 5%, and 10%), we also developed a spin-drying procedure (Supplementary video V1) to eliminate the ambient benchtop dry. After incubation with StabilCoat Plus^®^, chips affixed to a comb were attached to the rotating platter of a wafer polisher and spun at 500 rpm to remove liquid. The spin dry prevents liquid from puddling on the surface, while leaving behind sufficient stabilizer to maintain SA integrity. The exact ingredients comprising StabilCoat Plus^®^ are proprietary, but it is known that the mixture contains bovine protein and it likely contains a sugar similar to trehalose. The 5% and 10% StabilCoat Plus^®^ spin-dried surfaces were too thick for probes to diffuse through without interfering with each other, as seen in the images taken with the microarrayer overhead camera (Figure 3). Those surfaces also produced diffuse probe spots with poor morphology when imaged with AIR. The 1% and 5% nitrogen-dried surfaces were thin enough for probes to dissolve through and maintain crisp borders (Figure 3) but lacked good probe morphology as seen in the AIR images. This suggests that the surface had not been sufficiently protected from degradation. Optimum conditions were found for either 1% StabilCoat Plus^®^ with a spin-dry, or 10% StabilCoat Plus^®^ with nitrogen dry. Since the spin-dry procedure required 10x less StabilCoat Plus^®^ than nitrogen drying to achieve optimal surface protection, all further experiments were conducted using spin-dried chips coated with 1% StabilCoat Plus^®^. However, it is useful to know that sufficient protection of a streptavidin film can be achieved with different drying conditions just by tuning the concentration of StabilCoat Plus^®^, an observation that could translate to the development of other sensor systems requiring a stable streptavidin film. 

### 3.4. Optimization of Biotin Linker and Streptavidin Concentration

It has been reported that a high density of biotin molecules on a planar surface can sterically hinder the amount of streptavidin that binds to the surface. A high surface density of streptavidin can also inhibit capture of biotinylated probes [8]. To address these issues, we tested different ratios of amine-PEG11-biotin to amine-PEG4-methyl (as a spacer molecule), and different concentrations of streptavidin for chip surface preparation. These chips were protected with StabilCoat Plus^®^ and dried following the optimized protection protocol prior to microarray spot deposition of biotinylated probes. The amount of probe retained on the chip surface was used as an indicator of ideal surface conditions, as quantified by AIR as the thickness difference between the probe spot and the background of the chip. Two probe types, anti-FITC and thrombin 29-mer aptamer (discussed further in the next section), were analyzed (Figure S4a). Two chip surface conditions resulted in the greatest probe deposition for both probe types: 20% biotin:80% methyl or 40% biotin:60% methyl and 80 µg/mL streptavidin. Since an increase in probe deposition does not always result in increased target detection, we also exposed the chips to thrombin protein and measured the thrombin protein build onto the 29-mer probe spots with AIR (Figure S4b). Taking these data together, the 40% biotin:60% methyl and 80 µg/mL streptavidin surface was deemed optimal for both probe deposition and target detection. Further experiments testing variation in the amount of streptavidin used suggested that a 30%biotin:70% methyl and 40 µg/mL streptavidin also worked well and reduced the amount of streptavidin required (Figure S4c). This protocol was used for all further experiments.

### 3.5. Target Detection with Biotin-Conjugated Aptamer Arrays

Aptamers are single stranded DNA or RNA oligonucleotides that fold into defined three-dimensional structures with specific and sensitive affinity for target proteins. Many aptamers have been selected and enriched from oligonucleotide libraries via the SELEX process [20,21], and are cataloged in the Aptagen “Apta-Index^TM^” database [10]. Once the aptamer sequence is known, it is simple and inexpensive to synthesize, and batch-to-batch variation is low [22]. Aptamers have been used as an alternative to antibodies as detection probes in a diagnostic context, and they have also been used therapeutically to alter biochemical pathways [23]. As an initial test of the biotin-streptavidin AIR surface for aptamer immobilization, we focused on aptamers for thrombin and TGFβ1. Thrombin aptamers have been well characterized and used in many sensing devices to detect thrombin, a critical protein for blood-clot formation [24]. Two different DNA aptamers have been identified that recognize thrombin: a 15-mer with a reported dissociation constant (K_D_) of ~100 nM, and a 29-mer with a reported K_D_ of ~0.5 nM [25,26]. TGFβ1 is a cytokine with important immune-regulating functions. A thioaptamer has been identified that recognizes TGFβ1 with a reported K_D_ of ~90 nM [27]. 

The two different thrombin aptamers (15-mer and 29-mer, each with a 20-mer poly-T spacer and 5′ biotin) were each arrayed onto the B-SA surface at concentrations of 10 µM, 50 µM, and 100 µM with six replicate spots at each concentration (Figure S5). Since these aptamer probes were deposited onto the surface of the AIR chip at a thickness near zero reflectivity, any increase in intensity is consistent with an increase in thickness due to a binding event between aptamer and thrombin. We found that the 29-mer was better able to detect thrombin protein than the 15-mer probe (Figure S5). When quantified, the 29-mer probe demonstrates an expected sigmoidal binding curve as thrombin concentration changes. However, we observed no significant differences in intensity from 15-mer probe spots as a function of thrombin concentration. The superiority of the 29-mer may be due to its tighter binding constant, and/or because its slightly larger molecular size results in an initial antireflective condition slightly to the right of the zero point, enabling improved binding detection on the AIR platform. The 15-mer may have been “stuck in the minimum” of the parabolic intensity vs. thickness curve [3]. Optimization of deposition conditions for the 15-mer could improve its performance. For the time being, we chose to continue with the 29-mer for further experiments. 

**Figure 4:**
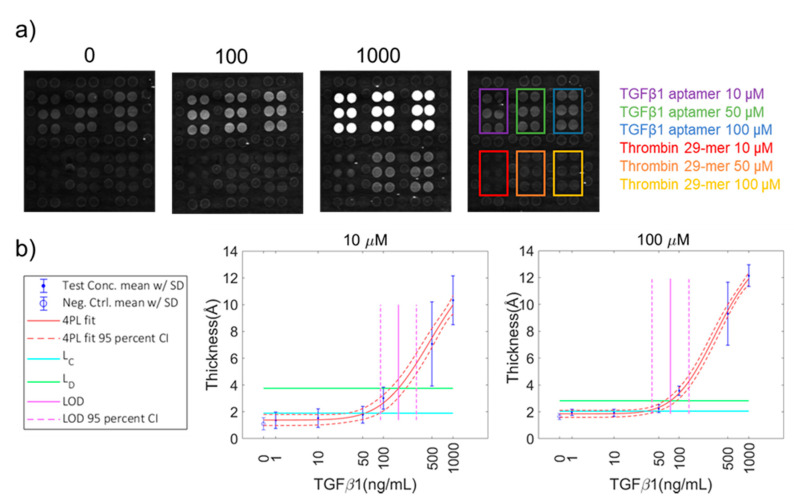
AIR detection of TGFβ1 using an aptamer probe. Representative array images of chips incubated in TGFβ1 protein at concentrations including 0 (control), 100 ng/mL, and 1000 ng/mL. All images were taken with a 500-ms exposure time. The baseline thickness of the TGFβ probes is greater than thrombin 29-mer probes, resulting in brighter spots in the 0 ng/mL image. As discussed in the introduction and methods, AIR is a differential measurement, and differences in baseline probe thickness are accounted for by the control chip. A map indicates the location of probes on each chip; probes are in six packs surrounded by normalizing probes (**a**). Plots of normalized thickness vs. target protein concentration were fitted with 4 parameter logistic curves and used to calculate limit of detection (LOD) for each protein concentration. Only the highest (100 µM) and lowest (10 µM) probe concentration plots are shown here. Error bars represent the standard deviation of all probe replicates (n ≥ 8) (**b**).

**Figure 5:**
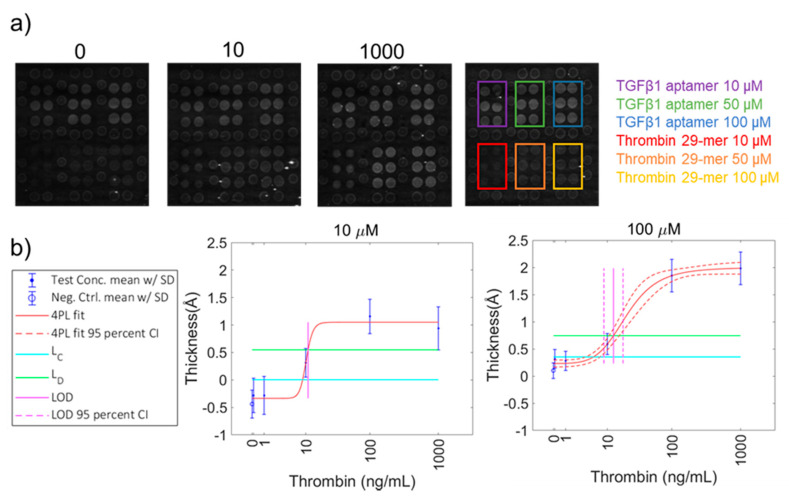
AIR detection of thrombin using a 29-mer aptamer. Representative array images of chips incubated in thrombin protein at concentrations varying from 0 (control) to 1000 ng/mL. All images were taken with a 500-ms exposure time. The baseline thickness of the TGFβ probes is greater than thrombin 29-mer probes, resulting in brighter spots in the 0 ng/mL image. As discussed in the introduction and methods, AIR is a differential measurement, and the difference in baseline probe thickness is accounted for by the control chip. A map indicates the location of probes on each chip; probes are in six packs surrounded by normalizing probes (**a**). Plots of normalized thickness vs. target protein concentration were fitted with 4 parameter logistic curves and used to calculate LOD for each protein concentration. Only the highest (100 µM) and lowest (10 µM) probe concentration plots are shown here. Error bars represent the standard deviation of all probe replicates (n ≥ 8) (**b**).

Next, we examined arrays incorporating both the thrombin 29-mer aptamer and an aptamer for TGFβ1 (Figure S6a). In the interest of determining whether probe deposition concentration affected the limit of detection (LOD) of target protein, we arrayed three different concentrations of each probe type onto the chip surface (10, 50, and 100 µM of aptamer). The chips were exposed to a serial dilution of target protein. In general, the TGFβ1 aptamer deposited onto the chip surface with more mass (greater initial thickness) than the thrombin 29-mer; therefore, the TGFβ1 aptamer probe spots are brighter than the thrombin 29-mer probe spots at the condition of 0 ng/mL added target. The chips were incubated in solutions of target TGFβ1 protein or thrombin protein separately; the target proteins were not mixed together. Both the Thrombin 29-mer and the TGFβ1 aptamer were able to detect their respective target protein in solution at decreasing concentrations of probe and antigen, with only minimal cross-reactivity. A small amount of nonspecifically bound TGFβ was observed on the 29-mer thrombin aptamer at the highest concentration (1µg/mL), but this did not occur at lower concentrations (Figure 4a). There was no visible cross-reactivity between the TGFβ1 probes and thrombin protein (Figure 5a). We observed no statistically significant binding of either TGFβ1 or thrombin proteins to the background.

The probe concentration did not greatly affect the overall shape of the dilution curve for most probe concentrations except for 10-µM thrombin 29-mer. Low probe deposition for this aptamer resulted in a low dynamic range of thrombin protein detection (Figure 5b). Additionally, the 4PL fit was poor for 10-µM thrombin 29-mer, which resulted in a large error on the calculation of the limit of detection (LOD) (Table 1). Use of the 100-µM probe spotting concentration provided a modest improvement in LOB and LOD for TGFβ1 over the 10 µM probe spotting concentration (32.2 and 53.7 ng/mL, respectively). The higher probe concentration might provide more consistent surface coverage and reduce error in the measurement. To compare the LODs of different assay conditions, a Student’s *t*-test of unequal variances comparing the calculated LODs [12] between TGFβ1 aptamer probe deposition concentrations returned p-values of <0.00001 for every pairing (Table S1). A comparison between the 50-µM and 100-µM thrombin 29-mer probe deposition concentrations returned the same significance. The degrees of freedom (df) were calculated by taking each assay as a whole, including every replicate at each sample dilution (n = 119). Such high df values contribute to low p-values. The differences in binding curve shape and LODs between thrombin and TGFβ1 reflect the difference in their reported binding constants.

### 3.6. Aptamer and Protein Mixed Probe Array

Having successfully demonstrated methods for the preparation and use of aptamer arrays, we next turned to fabrication of mixed arrays consisting of protein and aptamer probes on a single chip. Initial efforts to create these mixed arrays focused on printing biotin-conjugated antibodies onto the B-SA surface. Commercial biotinylated antibodies were tested, as well as antibodies biotinylated via amine-reactive chemistry in-house with an optimized procedure that aimed for 3–5 biotins per antibody. These biotin-conjugated antibodies adhered to the biotin-streptavidin surface well and produced clean arrays but were unable to detect their corresponding antigens. This failure to detect antigens was not a result of the biotin-conjugation process because binding ability was confirmed via dot blot. We hypothesize that the multiple biotins per antibody bind to multiple streptavidins on the B-SA chip surface in a manner that blocks the antigen recognition site of the antibodies. 

**Figure 6:**
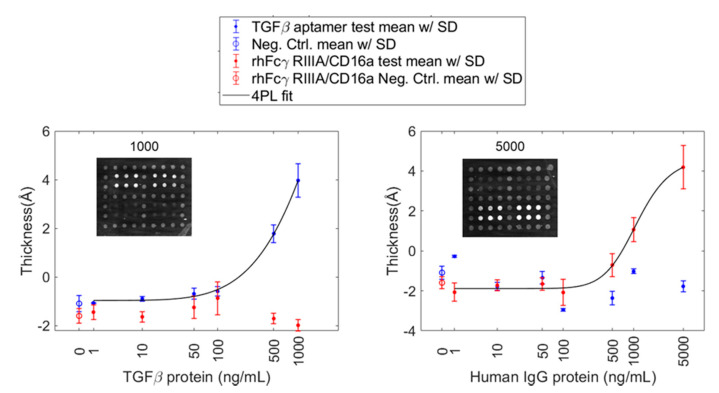
Performance of a dual aptamer-protein AIR array. Concentration-dependent response for arrays exposed to recombinant human TGF β1 and human IgG. Representative array images are shown in each case for 1000 ng/mL protein exposure. Plots of normalized thickness vs. target protein concentration were fitted with 4 parameter logistic curves. Error bars represent the standard deviation of all probe replicates (n ≥ 8).

To test this hypothesis, we purchased a recombinant protein Fcγ RIIIA/CD16a (F176) incorporating a single biotin “avi-tag”. The avi-tag is a 15-residue peptide that is a substrate for BirA, the *E. coli* biotin holoenzyme synthetase [28,29]. Proteins expressed with this tag are obtained with a single biotin. This protein probe was printed onto the B-SA chip alongside TGFβ1 aptamer to create a mixed aptamer-protein array (Figure S6b). The arrays were incubated separately in either TGFβ1 protein or human IgG. The mixed array was able to detect both proteins without any cross-reactivity (Figure 6). This suggests that a single biotin per probe molecule is important for retaining antigen recognition on this biotin-streptavidin surface and is a key factor in constructing functional mixed arrays.

## 4. Conclusions

Array-based sensor technologies are most useful when they can take advantage of a broad selection of capture probes. Here, we have demonstrated the first successful use of aptamer arrays for protein detection with the AIR biosensor platform. Successful immobilization of the aptamers required development of a stable biotin-streptavidin surface. In this context, we observed and characterized the degradation of the biotin-streptavidin layer upon drying and rewetting and developed a mitigation protocol using a coating of StabilCoat Plus^®^ that can be tuned depending on the drying method. As the biotin linker is attached to the surface via a stable (irreversible) covalent bond, we anticipate that optimized protocols developed here will be transferrable to other sensors in which a streptavidin layer is built up on a surface via a covalently attached biotin film. This knowledge may be useful in other contexts where this common biological pair is used for conjugation of molecules to planar surfaces at an air-water interface. It is very important to protect such a surface from the harsh drying and rewetting process, and it seems that a thin barrier is all that is needed. Finally, we have also demonstrated that it is possible to create mixed arrays of aptamer and protein using this surface. It should now be possible to deposit probes of any molecule with a single biotin label. The techniques used here to create and preserve the biotin-streptavidin layer may be useful in future work with probes that were previously occluded from use on the AIR platform.

## Figures and Tables

**Table 1:** Analytical performance for aptamer-based detection on AIR as a function of probe and probe deposition conditions. Each LOD, with the exception of the 10-µM probe concentration of Thrombin 29-mer because of the high error in the calculation, is statistically distinct based on a Student’s *t*-test of unequal variances.

Aptamer Probe	AIR Probe Concentration (µM)	Greatest Thickness Build from Probe Binding Target, Measured by AIR (Å)	LOB (ng/mL)	LOB (nM)	LOD + SE (ng/mL)	LOD (nM)	Reported K_D_ (nM)
Thrombin 29-mer	10	1.4	8.8	0.24	10.9 ± 271.5	0.30	0.5
Thrombin 29-mer	50	2.1	7.6	0.21	15.3 ± 0.1	0.43	0.5
Thrombin 29-mer	100	1.9	5.6	0.16	12.7 ± 0.1	0.35	0.5
Thrombin 15-mer	10, 50, 100	N/A	N/A	N/A	N/A	N/A	25–200
TGFβ1	10	9.2	53.7	2.1	165.3 ± 0.1	6.6	90
TGFβ1	50	10.5	62.1	2.5	135.3 ± 0.1	5.4	90
TGFβ1	100	10.5	32.2	1.3	74.5 ± 0.1	3.0	90

## Supplementary Materials

The following are available online at https://www.mdpi.com/1424-8220/20/20/5745/s1, Figure S1: Growth and protection of the biotin-streptavidin surface for biotin-probe deposition, Figure S2: Schematic definition of the calculation of normalized thickness (Å) in AIR, Figure S3: Surface degradation and different protective coating mixtures, Figure S4: Arrays on chip surfaces containing different ratios of biotinylated and non-biotinylated linkers and streptavidin concentrations, Figure S5: Detection of thrombin protein using two different aptamer probes (29-mer and 15-mer), Figure S6: Probe layout for AIR images in Figure 4 and Figure 5 (a), and Figure 6 (b), Table S1: Values used to calculate p-values for LOD comparisons, Video S1: Spin dry procedure.
