# Supplementary material for: A Stable Biotin-Streptavidin Surface Enables Multiplex, Label-Free Protein Detection by Aptamer and Aptamer-Protein Arrays Using Arrayed Imaging Reflectometry
Source: Sensors (Basel). 2020 Oct 10;20(20):5745. doi: 10.3390/s20205745 (PMC7650819; doi:10.3390/s20205745)
Supplement: Supplementary file 1 [file sensors-20-05745-s001.zip › sensors-949520-supplementary.pdf]

sensors MDPI

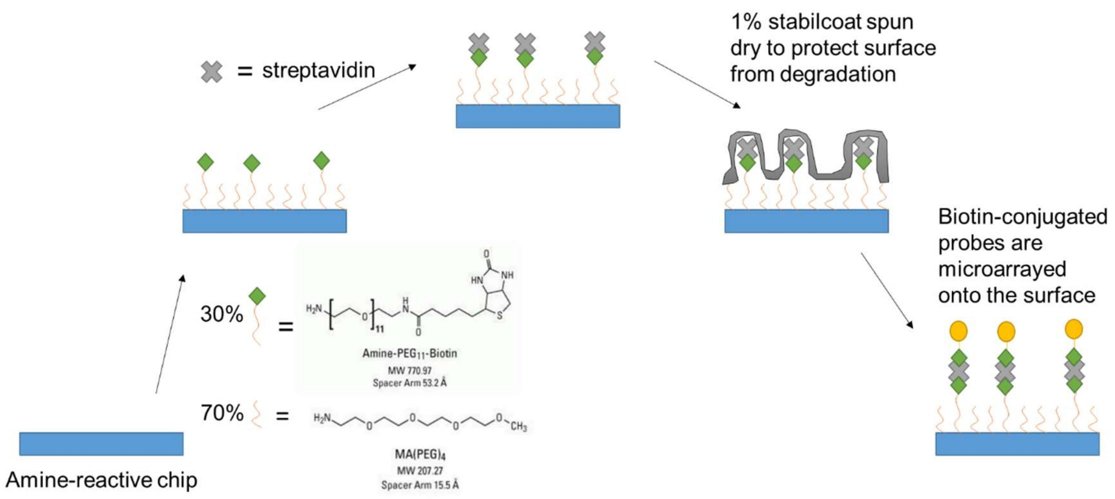

Figure S1. Growth and protection of the biotin-streptavidin surface for biotin-probe deposition.

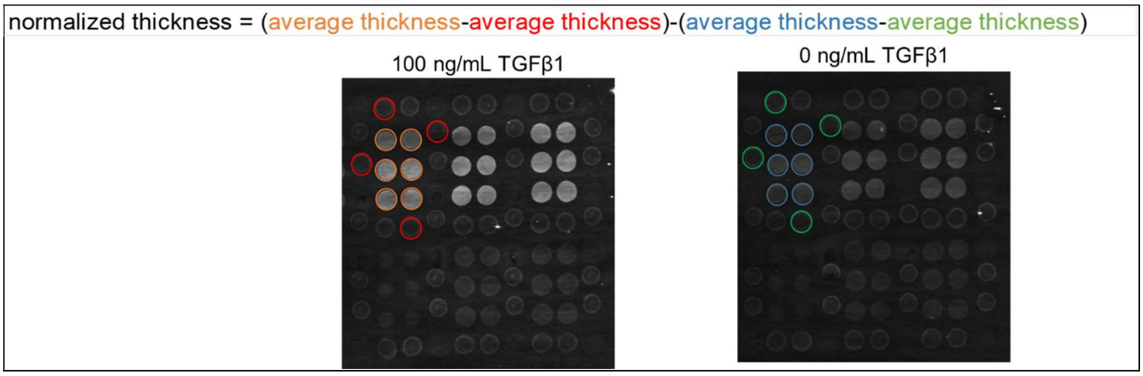

Figure S2. Schematic definition of the calculation of normalized thickness (Å) in AIR.

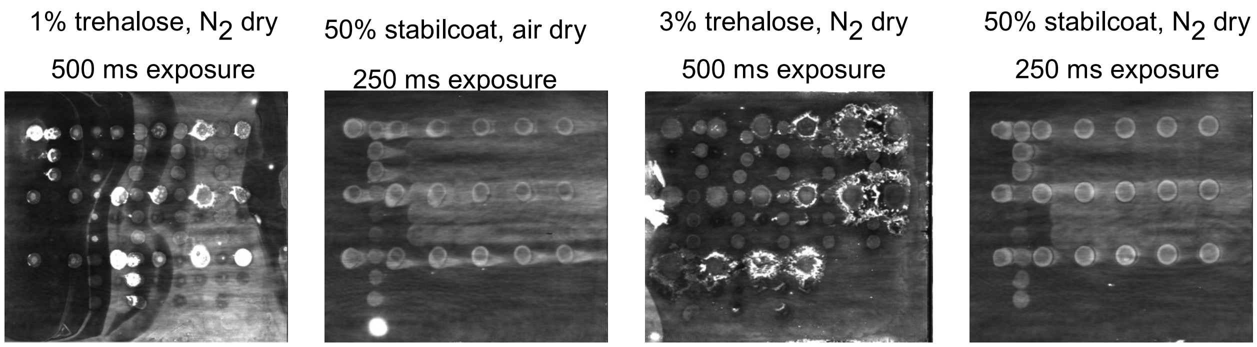

Figure S3. Surface degradation and different protective coating mixtures dried under a stream of nitrogen gas or air dried on the laboratory benchtop. Trehalose alone resulted in unacceptable spot and background artifacts. 50% stabilcoat was too thick for probes to dissolve through and reach the streptavidin surface below without significant lateral diffusion.

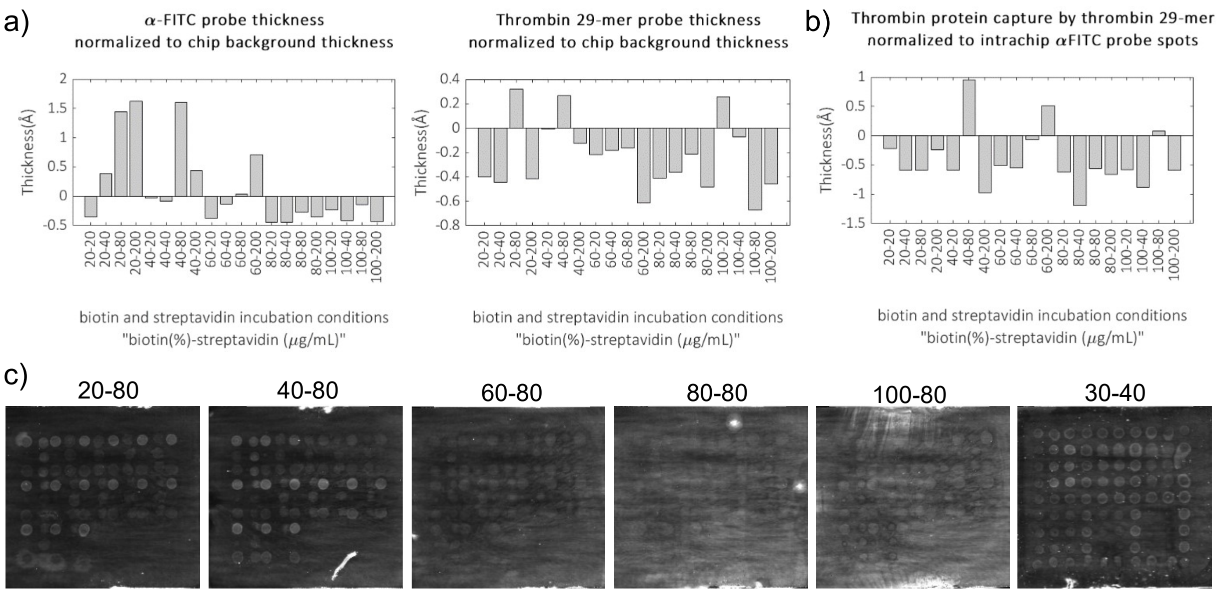

Figure S4. Arrays on chip surfaces containing different ratios of biotinylated and non-biotinylated linkers and streptavidin concentrations. The first number indicates percentage of biotinylated linker attached to the substrate surface. The second number indicates concentration of streptavidin incubated with the biotinylated surface (µg/mL). Higher concentrations of biotin produced thicker streptavidin layers as measured by AIR (for example, 1422.3 Å for 100–80 vs. 1419.7 Å for 20–80). Initial measurements of probe deposition thickness via AIR indicated that 20% or 40% biotin linker and 80 µg/mL streptavidin was ideal and led to greatest amount of probe deposited compared to the background (**a**), which is also seen in the AIR images (all taken with a 500 ms exposure time) (**c**). Measurements of target capture via AIR indicated that the 40% biotin linker with 80 µg/mL streptavidin condition led to the greatest amount of target thrombin captured by aptamer (**b**). Probe deposition from later experiments using a 30% biotin linker and 40 µg/mL streptavidin was acceptable and comparable to the 20% biotin : 80% methyl and 40% biotin : 60% methyl conditions (**c**).

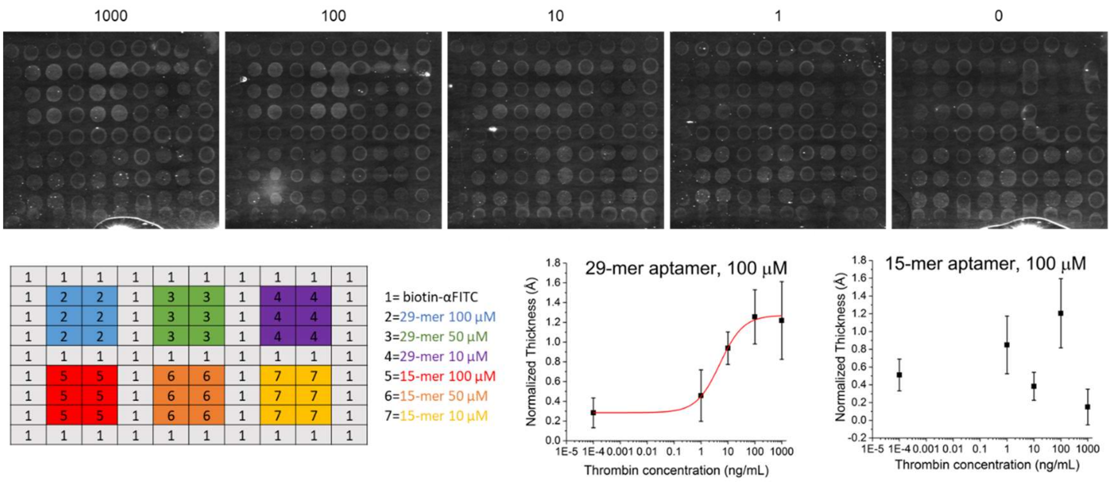

Figure S5. Detection of thrombin protein using two different aptamer probes (29-mer and 15-mer). Representative array images of chips incubated in thrombin protein in concentrations varying from 0 (control) to 1000 ng/mL. Thicknesses are measured by AIR and normalized to the biotin-αFITC intra-chip control spots, plotted, and fit with a nonparametric logistic curve. Error bars are the standard deviation of replicate probe spots over two chips (n = 12).

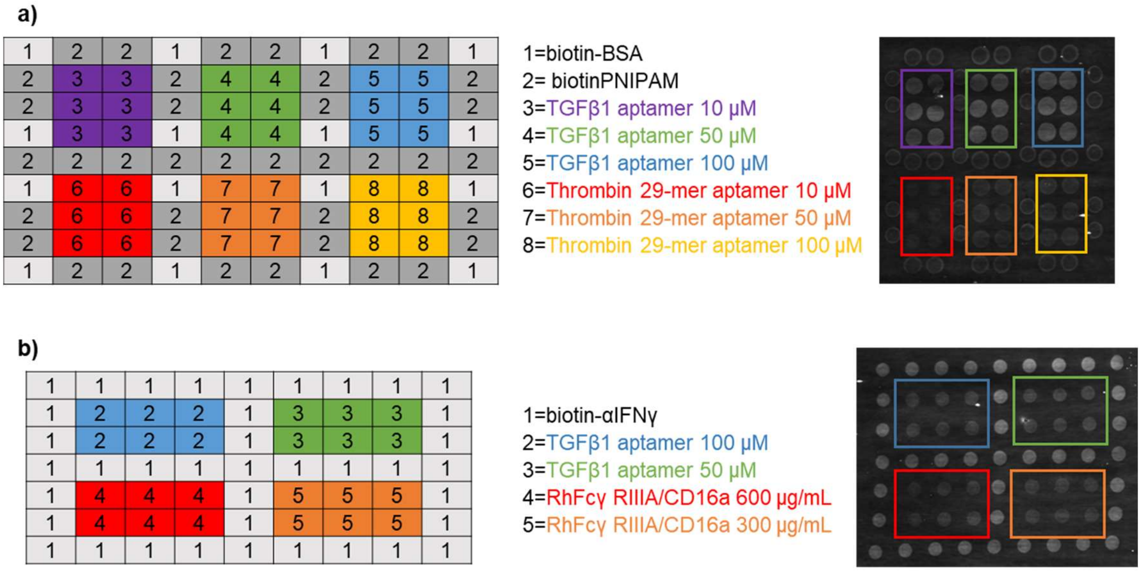

Figure S6. Probe layout for AIR images in figures 4 and 5 (**a**), and figure 6 (**b**).

Table S1. Values used to calculate p-values (all $p < 0.00001$) for significant difference in resulting LOD of the assay according to probe deposition concentration.

	probe concentrations	t-statistic	degrees of freedom
**TGFβ1**	10 µM vs 50 µM	182.6	215.4
	10 µM vs 100 µM	484.8	229.7
	50 µM vs 100 µM	363.2	212.0
**Thrombin 29-mer**	50 µM vs 100 µM	23.7	225.1
